# *Nuf2* Is a Prognostic-Related Biomarker and Correlated With Immune Infiltrates in Hepatocellular Carcinoma

**DOI:** 10.3389/fonc.2021.621373

**Published:** 2021-03-09

**Authors:** Xingwei Xie, Shanshan Jiang, Xiang Li

**Affiliations:** ^1^ College of Plant Protection, Henan Agricultural University, Zhengzhou, China; ^2^ Key Laboratory of Forensic Toxicology of Herbal Medicines, Guizhou Education Department, School of Basic Medicine, Guizhou University of Traditional Chinese Medicine, Guiyang, China

**Keywords:** *Nuf2*, hepatocellular carcinoma, biomarkers, tumor immunity, prognosis

## Abstract

*Nuf2* participates in the regulation of cell apoptosis and proliferation by regulating the binding of centromere and spindle microtubules to achieve the correct separation of chromosomes. Previous reports have suggested that *Nuf2* may play a role in various human cancers. However, the mechanism and function of *Nuf2* in the development of Hepatocellular carcinoma (HCC) remains uncertain. This study investigated the prognostic potential of *Nuf2* and its relation with immune cell infiltration in HCC. *Nuf2* expression in tumor cells was examined using the TIMER and Oncomine databases, and its prognostic potential was assessed *via* the Kaplan-Meier plotter and GEPIA databases. The relationships between *Nuf2* and tumor immune infiltration were analyzed using TIMER. The relationships between *Nuf2* and biomarkers of tumor immune infiltration were analyzed using TIMER and GEPIA. Here we revealed that *Nuf2* expression increased in tumor tissues containing HCC, and this correlated with poor relapse-free survival, disease-specific survival, progression-free survival, and overall survival in patients with HCC regardless of grades, genders, races, drinking behaviors and other clinical factors. Additionally, high expression of *Nuf2* was positively correlated with differential immune cell infiltration and various immune biomarkers. Our work demonstrated that *Nuf2* could be a potential prognostic biomarker and could be related to tumor immune cell infiltration in HCC.

## Introduction

Hepatocellular carcinoma (HCC), caused by factors such as alcoholic hepatitis, chronic hepatitis B and hepatitis C infections, and nonalcoholic fatty liver disease, has become one of the most commonly diagnosed cancers (1–4). HCC is not only highly malignant and difficult to treat, but also has poor prognosis and high relapse rates, resulting in overall 5-year survival rates of only 5%–9% (5). Early identification of at-risk patients, exploration of new biomarkers and therapeutic targets for diagnosis, and in-depth understanding of the molecular pathogenesis of HCC are prerequisites for controlling this disease.

Nuf2, also known as CDCA1, is a key element of the Ndc80/Nuf2 complex that is required for the formation of stable kinetochore-microtubule attachments and chromosome alignment during mitosis. *Nuf2* participates in the regulation of cell apoptosis and proliferation by regulating the binding of centromere and spindle microtubules to achieve the correct separation of chromosomes (6, 7). *Nuf2* can be cleaved into alternative splice variants and expressed differentially between tumor tissues and the corresponding normal tissues (8). *Nuf2* promotes the tumorigenesis and tumor development and is highly expressed in various human cancers, including serous adenocarcinoma, renal cell carcinoma, cholangiocarcinoma, and colorectal, lung, ovarian, gastric, and bladder cancers (8–13). Knockout of the *Nuf2* gene significantly delayed cell growth and increased apoptosis in ovarian, stomach, and colorectal cancer cell lines (9, 10, 12). High *Nuf2* expression has been reported to correlate with poor prognosis in non-small cell carcinoma patients (10). Additionally, *Nuf2* can also function as a potential biomarker in human tumor diagnosis and immunotherapy (10). These findings strongly imply a potential function of *Nuf2* in tumorigenesis. However, the mechanism and role of *Nuf2* in the development of HCC remains uncertain.

This study investigated the prognostic potential of *Nuf2* and its relation with immune cell infiltration in HCC. *Nuf2* expression in tumor cells was examined using the TIMER and Oncomine databases, and its prognostic potential was assessed *via* the Kaplan-Meier plotter and GEPIA databases. The relationships between *Nuf2* and tumor immune infiltration were analyzed using TIMER. The relationships between *Nuf2* and biomarkers of tumor immune infiltration were analyzed using TIMER and GEPIA. Here we revealed that the expression level of *Nuf2* was significantly increased in HCC, and was correlated with the prognosis of HCC patients. We suggest that *Nuf2* has the potential to be a diagnostic gene in hepatocarcinogenesis and prognostic biomarkers for HCC patients.

## Materials and Methods

### Oncomine Analysis

Oncomine platform (https://www.oncomine.org/) is a publicly accessible online tumor related-gene microarray database, which collects the related gene expression profiles and relevant clinical information. The transcriptional levels of *Nuf2* in different tumors and corresponding normal tissues were analyzed by Oncomine. The expression levels were considered different significantly when fold change is greater than 1.5, with *P*-value < 0.001. We set the threshold value of gene rank to “top 10%” and the data type to “all” (14).

### Kaplan–Meier Plotter Analysis

The Kaplan-Meier plotter was used for analyzing the relationship between survival rate and *Nuf2* expression in breast, gastric, liver, lung, and ovarian cancers based on two parameters, namely hazard ratios (HR) and log-rank *P*-values (15). We performed survival analysis with the parameters of Group Cutoff: Median; Hazards Ratio: Yes; 95% Confidence Interval: Yes.

### TIMER Analysis

The TIMER database (http://timer.comp-genomics.org/) is a consolidated database to analyze the immune infiltration in different tumor types. Information from 32 types of tumors with more than 10,000 samples from TCGA database was used for immune infiltration analysis *via* the TIMER database. TIMER ascertains the abundance of tumor infiltrates based on gene expression (16). The correlations between *Nuf2* expression and immune cell infiltration levels in different tumors were analyzed according to biomarker gene expression in tumors. The biomarker genes of tumor-infiltrating immune cells including B cells, CD8+T cells, dendritic cells, T cells (general), TAMs, M1 macrophages, M2 macrophages, monocytes, neutrophils, natural killer cells, T-helper cells (Th), Tregs, follicular helper T cells (Tfh), and exhausted T cells were investigated in this study (17, 18).

### GEPIA Analysis

GEPIA (http://gepia.cancer-pku.cn/index.html) is a web server for analyzing the RNA sequencing expression data from the TCGA and the GTEx projects, using a standard processing pipeline (19). We analyzed the correlation between *Nuf2* and different immune cell biomarkers *via* GEPIA. The correlation coefficient was determined by the Spearman method with default parameters.

## Results

### The Expression of *Nuf2* in HCC and Other Cancers

**Figure 1:**
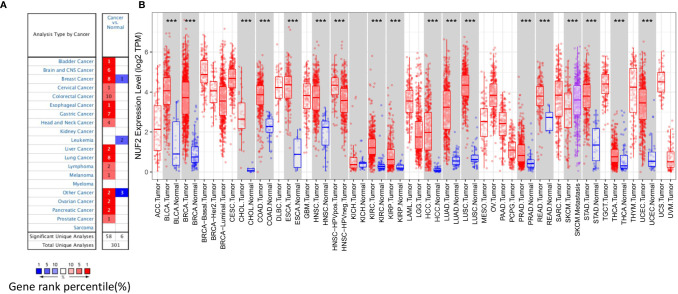
*Nuf2* expression in different human tumor cell types. **(A)** *Nuf2* expression in cancer tissue types compared to that in normal tissues (data from Oncomine). **(B)** Level of *Nuf2* expression in various cancers (data from TCGA *via* TIMER) (***P<0.001).

Analysis using the Oncomine platform revealed a significant increase in the transcription level of *Nuf2* in a variety of cancerous tissues compared to normal tissues, including liver and other 14 types of cancers (bladder, brain and central nervous system, breast, cervical, colorectal, esophageal, gastric, head and neck, lung, lymphoma, melanoma, ovarian, pancreatic, and prostate cancers) (**Figure 1A**). Additionally, analysis of TCGA RNA-seq data in TIMER database, we found consistent results, i.e. the expression of *Nuf2* in HCC was significantly higher than that in normal tissue, as well as other 16 tumor tissues (**Figure 1B**), suggesting that *Nuf2* may play a role in tumorigenesis, especially in HCC, and has the potential to be a diagnostic gene for liver cancer and other cancers.

### Prognostic Potential of *Nuf2* Expression in Hepatocellular Carcinoma

Overall survival (OS), disease specific survival (DSS), relapse free survival (RFS) and progression free survival (PFS) are four common prognostic monitoring indexes. As the name suggests, these four indicators can basically summarize the prognosis and survival of cancer patients. Using the Kaplan-Meier plotter, we found that the expression level of *Nuf2* gene was significantly correlated with the prognostic survival rate. For example, poor first progression survival and overall survival in lung cancer; poor post progression survival, overall survival and progression free survival in ovarian cancer; poor post progression survival, first progression survival and overall survival in gastric cancer; and poor relapse-free survival in breast cancer (**Supplementary Figure S1**).

**Figure 2:**
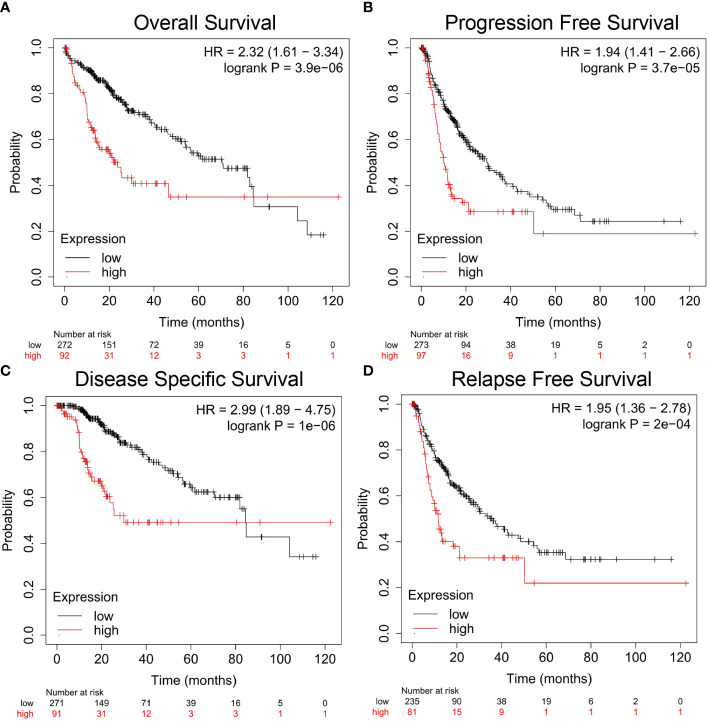
Correlation analysis between *Nuf2* expression and prognostic survival in HCC patients *via* Kaplan-Meier plotter analysis. **(A)** Overall survival, n = 364; **(B)** Progression-free survival, n = 370; **(C)** Disease-specific survival, n = 362; **(D)** Relapse-free survival, n = 316.

In particular, for HCC, we found that the DSS (**Figure 2A**, HR=2.99, 95% CI=1.89 to 4.75, *P*=1e-06), PFS (**Figure 2B**, HR=1.94, 95% CI=1.41 to 2.66, *P*=3.7e-05), OS (**Figure 2C**, HR=2.32, 95% CI=1.61 to 3.34, *P*=3.9e-06), and RFS (**Figure 2D**, HR=1.95, 95% CI=1.36 to 2.78, *P*=2e-04) were significantly reduced when the expression level of *Nuf2* was high, indicating that active transcription of *Nuf2* might cause health risks, and these genes could be potential prognostic biomarkers for HCC patients.

### Relationship Between *Nuf2* Expression and Clinical Features in Hepatocellular Carcinoma Patients

The correlation between *Nuf2* expression and various clinical features in HCC patients was evaluated *via* the Kaplan-Meier plotter. High expression of *Nuf2* was associated with poor OS and PFS for HCC patients regardless of genders (female and male), races (white and Asian), HCC grades or alcohol consumption. Particularly, high *Nuf2* expression was correlation with poor OS and PFS in grades 1 to 3 in HCC patients, indicating that high *Nuf2* expression might be harmful to the prognosis of HCC patients (**Table 1**). Notably, *Nuf2* expression was only associated with survival in the absence of hepatitis virus, but not in the presence of hepatitis virus. Interestingly, when there was vascular invasion in HCC, *Nuf2* expression and PFS show a significant negative correlation; while when there is no vascular invasion, *Nuf2* and OS show a significant negative correlation (**Table 1**). The differences in clinical features suggest that the application of *Nuf2* as an indicator gene should be combined with the patient’s condition.

**Table 1:** Correlation of *Nuf2* expression and prognosis in HCC with diverse clinicopathological factors by Kaplan-Meier plotter.

Clinicopathological factors		Overall survival (n = 364)			Progression-free survival (n = 366)		
		N	Hazard ratio	*P*-value	N	Hazard ratio	*P*-value
**Sex**	Female	118	2.37(1.27–4.42)	**5.3E-03**	120	1.69(1.01–2.82)	**0.042**
	Male	246	3.61(1.91–6.84)	**2.4E-05**	246	1.96(1.33–2.9)	**5.6E-04**
**Grade**	1	55	7.25(2.43–21.61)	**6.4E-05**	55	2.56(1.05–6.2)	**0.033**
	2	174	2.01(1.2–3.36)	**7.1E-03**	175	2.32(1.48–3.61)	**1.4E-04**
	3	118	3.05(1.64–5.67)	**2.3E-04**	119	3.27(1.19–5.61)	**5.5E-06**
	4	12			12		
**Vascular invasion**	Yes	90	1.96(0.73–5.26)	0.17	91	2.44(1.31–4.55)	**3.9E-03**
	None	203	2.17(1.23–3.82)	**6.1E-03**	204	1.44(0.88–2.38)	0.15
**Race**	White	181	2.11(1.22–3.64)	**5.9E-03**	183	1.96(1.31–2.94)	**8.6E-04**
	Asian	155	5.63(2.99–10.61)	**2.4E-09**	155	2.81(1.71–4.6)	**2E-05**
**Alcohol consumption**	yes	115	3.01(1.4–6.46)	**3.1E-03**	115	2.92(1.69–5.05)	**6.3E-05**
	none	202	2.66(1.63–4.34)	**4.5E-05**	204	2.03(1.31–3.13)	**1.2E-03**
**Hepatitis virus**	Yes	150	1.88(0.98–3.63)	0.055	152	1.54(0.93–2.57)	0.092
	None	167	3.05(1.85–5.02)	**4.5E-06**	167	3.06(1.92–4.87)	**8.5E-07**

Bold values indicate P < 0.05.

### Relationship Between *Nuf2* Expression and Immune Cell Infiltration in Hepatocellular Carcinoma

**Figure 3:**
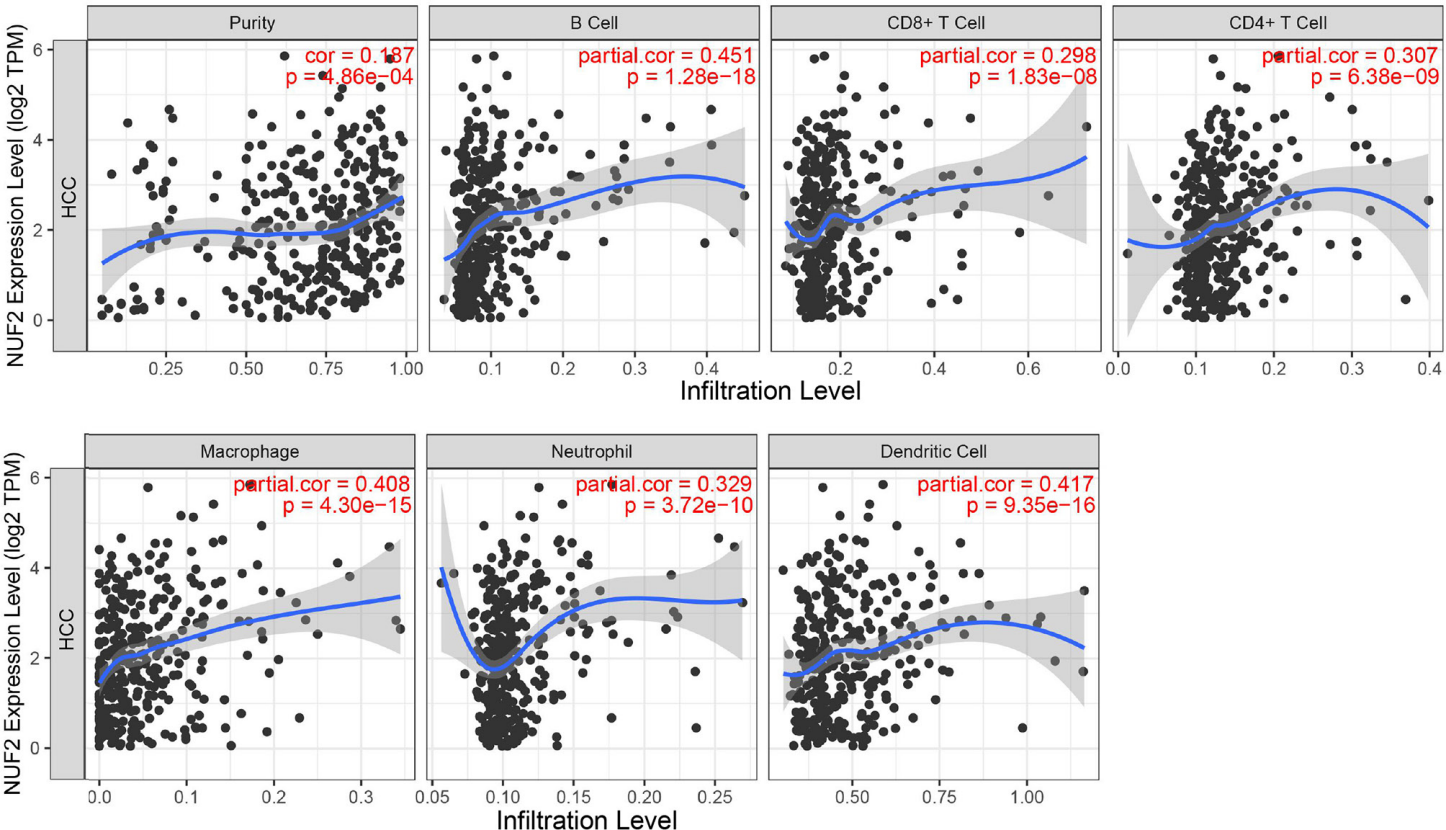
Correlation between *Nuf2* expression and immune cell infiltration levels in HCC tissues analyzed *via* TIMER (n = 371).

TIMER was used to investigate the correlation between the expression of *Nuf2* and infiltration levels of immune cells. In HCC, on the whole, high *Nuf2* transcripts were associated with high immune cell infiltration (**Figure 3**). Specifically, *Nuf2* expression was positively correlated with infiltration of B cells (r=0.451, *P=*1.28e-18); DCs (r=0.417, *P=*9.35e-16); macrophages (r=0.408, *P=*4.30e-15); neutrophils (r=0.329, *P=*3.72e-10); CD4+ T cells (r=0.307, *P=*6.38e-09); and CD8+ T cells (r=0.298, *P=*1.83e-08) in HCC (**Figure 3**).

### Correlation Between *Nuf2* Expression and Biomarkers of Different Immune Cell Subsets

The association between *Nuf2* expression and tumor-infiltrating immune cell status was investigated based on immune biomarker gene expression levels in HCC. Immune cells in HCC tissues contained dendritic cells, B cells, monocytes, CD4+ T cells, natural killer cells (NKs), CD8+ T cells, neutrophils, M1 macrophages, M2 macrophages, and tumor-associated macrophages (TAMs). Furthermore, different T cell subsets, including T-helper 17 (Th17), T-helper 2 (Th2), T-helper 1 (Th1), exhausted T cells, follicular helper T cells (Tfh), and regulatory T cells (Tregs) were analyzed. Analysis by GEPIA and TIMER indicated a significant positive correlation between *Nuf2* expression and most of biomarkers expression in immune cells in HCC (**Supplementary Figure S2** and **Table 2**).

**Table 2:** Correlation analysis between *Nuf2* and biomarker genes of immune cells in HCC.

Description	Gene markers	TIMER		GEPIA	
		Cor	FDR	Cor	FDR
CD8+ T cell	CD8A	0.272	**6.40E-07**	0.16	**5.29E-03**
	CD8B	0.244	**8.26E-06**	0.13	**1.85E-02**
T cell (general)	CD3D	0.367	**8.53E-12**	0.23	**2.73E-05**
	CD3E	0.326	**2.03E-09**	0.15	**6.10E-03**
	CD2	0.321	**3.27E-09**	0.17	**3.59E-03**
B cell	CD19	0.372	**5.19E-12**	0.30	**4.48E-08**
	CD79A	0.291	**9.49E-08**	0.15	**7.01E-03**
Monocyte	CD86	0.418	**4.76E-15**	0.24	**1.52E-05**
	CD115	0.246	**7.28E-06**	0.11	5.90E-02
TAM	CCL2	0.154	**5.19E-03**	0.027	6.43E-01
	CD68	0.283	**2.25E-07**	0.16	**4.43E-03**
	IL10	0.321	**3.26E-09**	0.11	5.48E-02
M1 Macrophage	iNOS	0.028	6.20E-01	0.026	6.42E-01
	IRF5	0.437	**2.42E-16**	0.40	**1.48E-13**
	COX2	0.207	**1.65E-04**	0.073	2.01E-01
M2 Macrophage	CD163	0.136	**1.35E-02**	-0.012	8.34E-01
	VSIG4	0.170	**2.33E-04**	0.043	4.40E-01
	MS4A4A	0.194	**3.96E-04**	0.044	4.37E-01
Neutrophils	CD66b	0.097	7.74E-02	0.07	2.21E-01
	CD11b	0.369	**7.51E-12**	0.27	**8.85E-07**
	CCR7	0.224	**4.46E-05**	0.067	2.41E-01
Natural killer cell	KIR2DL1	-0.045	4.18E-01	0.000	1.00
	KIR2DL3	0.211	**1.18E-04**	0.15	**6.79E-03**
	KIR2DL4	0.232	**2.33E-05**	0.23	**2.75E-05**
	KIR3DL1	0.024	6.53E-01	-0.054	3.47E-01
	KIR3DL2	0.148	**7.21E-03**	0.15	**6.47E-03**
	KIR3DL3	0.055	3.28E-01	0.11	**4.60E-02**
	KIR2DS4	0.055	3.28E-01	0.049	3.90E-01
Dendritic cell	HLA-DPB1	0.258	**2.57E-06**	0.12	**2.71E-02**
	HLA-DQB1	0.231	**2.58E-05**	0.083	1.41E-01
	HLA-DRA	0.295	**6.94E-08**	0.16	**5.66E-03**
	HLA-DPA1	0.256	**2.87E-06**	0.11	**4.28E-02**
	BDCA-1	0.202	**2.29E-04**	0.097	8.31E-02
	BDCA-4	0.224	**4.47E-05**	0.16	**5.46E-03**
	CD11c	0.442	**1.28E-16**	0.28	**3.12E-07**
Th1	T-bet	0.180	**1.05E-03**	0.062	2.83E-01
	STAT4	0.301	**3.48E-08**	0.23	**3.82E-05**
	STAT1	0.414	**8.51E-15**	0.35	**1.02E-10**
	IFN-γ	0.337	**5.13E-10**	0.25	**7.87E-06**
	TNF-α	0.368	**8.41E-12**	0.22	**7.54E-05**
Th2	GATA3	0.290	**1.06E-07**	0.16	**5.48E-03**
	STAT6	0.107	5.22E-02	0.15	**7.98E-03**
	STAT5A	0.277	**3.89E-07**	0.21	**1.74E-04**
	IL13	0.113	**4.03E-02**	0.13	**2.29E-02**
Tfh	BCL6	0.182	**9.58E-04**	0.18	**1.49E-03**
	IL21	0.151	**2.48E-03**	0.16	**5.45E-03**
Th17	STAT3	0.160	**3.81E-03**	0.12	**4.08E-02**
	IL17A	0.121	**2.80E-02**	0.089	1.14E-01
Treg	FOXP3	0.240	**1.19E-05**	0.12	**3.11E-02**
	CCR8	0.463	**5.47E-18**	0.35	**1.27E-10**
	STAT5B	0.244	**8.26E-06**	0.27	**1.25E-06**
	TGFβ	0.347	**1.50E-10**	0.22	**9.32E-05**
T cell exhaustion	PD-1	0.407	**2.26E-14**	0.30	**5.31E-08**
	CTLA4	0.460	**5.58E-18**	0.33	**1.36E-09**
	LAG3	0.344	**2.14E-10**	0.24	**1.32E-05**
	TIM-3	0.433	**4.18E-16**	0.25	**5.36E-06**
	GZMB	0.154	**5.12E-03**	0.05	3.86E-01
	TOX	0.276	**4.24E-07**	0.18	**1.93E-03**
	TIGIT	0.409	**1.93E-14**	0.26	**2.95E-06**

HCC, hepatocellular carcinoma; TAM, tumor-associated macrophage; Th, T helper cell; Tfh, Follicular helper T cell; Treg, regulatory T cell; Cor, R value of Spearman’s correlation. FDR, false discovery rate corrected P value. Bold values indicate FDR < 0.05.

Through the analysis of TIMER and GEPIA databases, a notable positive correlation was found between *Nuf2* expression and specific immune cell biomarkers, namely CD8+ T cell biomarkers (*CD8A*, *CD8B*), T cell (general) biomarkers (*CD2*, *CD3E*, *CD3D*), B cell biomarkers (*CD19*, *CD79A*), Monocyte biomarkers (*CD86*), TAM biomarkers (*CD68*), M1 Macrophage biomarkers (*IRF5*), Neutrophil biomarkers (*CD11b*), NK biomarkers (*KIR2DL3*, *KIR2DL4*, *KIR3DL2*), DC biomarkers (*CD11c*, *BDCA-4*, *HLA-DRA*, *HLA-DQB1*, *HLA-DPB1*, *HLA-DPA1*), Th1 biomarkers (*TNF-α*, *IFN-γ*, *STAT1*, *STAT4*), Th2 biomarkers (*IL13*, *GATA3*, *STAT5A*), Tfh biomarkers (*BCL6*, *IL21*), Th17 biomarkers (*STAT3*), Treg biomarkers (*TGF-β*, *STAT5B*, *CCR8*, *FOXP3*), T-cell exhaustion biomarkers (*TIGIT*, *TOX*, *TIM-3*, *LAG3*, *CTLA4*, *PD-1*).

## Discussion

According to the 2018 global cancer statistics report, the incidence and mortality rates of HCC ranked sixth and fourth, respectively (20). Although surgical resection, tumor vascular embolization, and radiofrequency ablation can improve survival rates, the probability that most patients will eventually encounter the invasion or progression of liver cancer is high, and the prognosis is usually poor (21–23). Immune escape, invasion, and metastasis further reduce the long-term survival rate of HCC patients (24). Through a series of bioinformatics analysis with the publicly accessible online databases, we investigated the expression levels of *Nuf2* in HCC and corresponding normal tissues, and the effect of *Nuf2* expression on survival of prognosis and immune cell infiltration. We showed that *Nuf2* expression increased in tumor tissues containing HCC, and this correlated with poor relapse free survival, disease specific survival, progression free survival, and overall survival in patients with HCC regardless of grades, genders, races, drinking behaviors and other clinical factors. Additionally, high expression of *Nuf2* was positively correlated with differential immune cell infiltration and various immune biomarkers. Our works demonstrated that *Nuf2* could be a potential diagnostic gene in hepatocarcinogenesis and prognostic biomarkers for HCC patients.

In the course of tumorigenesis and development, due to the post-transcriptional regulation mediated by small non-coding RNAs such as miRNA and lncRNA, the transcription level of mRNA sometimes inconsistent with the final protein expression. However, mRNA was still selected as our main research object in this study, because many physiological and biochemical interactions take place at the mRNA level. In addition, compared with protein, the transcriptional alterations of mRNA can be detected on a large scale by simple operation and low cost. To clarify the role of *Nuf2* in HCC, by multiple database analysis we revealed that *Nuf2* was highly expressed in HCC tissue, indicating that *Nuf2* has potential as a diagnostic gene for the occurrence and development of HCC. Similarly, *Nuf2* has been proved to be highly expressed in many other cancer types, indicating its wide applicability and functional conservation. However, there was no significant up-regulation of *Nuf2* was found in leukemia, suggesting that it is necessary to distinguish the types of cancer when they were used as diagnostic genes.

According to previous reports, *Nuf2*, also known as *CDCA1*, is mainly responsible for regulating cell mitosis (25). Down-regulation of *Nuf2* expression can inhibit the proliferation of tumor cells, while over expression of *Nuf2* is associated with poor prognosis (6, 13). This is consistent with the results of our study. In particular, there is a significant negative correlation between *Nuf2* and prognostic survival of OS, DSS, RFS, and PFS, and this correlation is generally applicable to HCC patients with different clinical conditions, indicating that high *Nuf2* expression may be one of the causes of poor prognosis. Cancer patients need careful observation after treatment, and *Nuf2* may be used, to some extent, as a prognostic marker to reduce the risk of recurrence.

There has been considerable progress in immune therapy for cancer in recent years, and people have begun to pay attention to the effects of the immune system in tumorigenesis and development (26). The study of tumor microenvironment is an active research field of tumor diagnosis, treatment targets, and prognostic biomarkers (27). Many subtypes of immune cells, for example of Th1, Th2, and tumor-associated macrophages (TAMs) have been reported in tumor microenvironment (28). *Nuf2* showed a significant positive correlation with various immune cells, indicating that Nuf2-mediated hepatocarcinogenesis might mobilize the activity of these immune cells and make them play an anti-tumor role. Macrophage was divided into M1 macrophage and M2 macrophage. M1 macrophages are mainly related to the recognition and attack of tumor cells, while M2 macrophages are related to tumor progression and immunosuppression (29–31). In this study we revealed that *Nuf2* expression was related to the biomarker genes of M1 macrophages, not M2 macrophages. We therefore hypothesized that *Nuf2* might not be involved in the mechanism of tumorigenesis mediated by M2 macrophage, but mainly play the anti-tumor role *via* M1 macrophages pathway. Our findings, to a certain extent, indicated the future research direction.

*Nuf2* was positively associated with the biomarkers of T-cell exhaustion (*PD-1*, *CTLA4*, *TIM-3*, *LAG3*, *TOX*, *TIGIT*). *TIM-3* is a T-cell suppressor molecule that can cause CD8+T cell (exhausted CD8+ T cells, TEX) to fail in chronic conditions and tumors (32). *TOX* is one of the most common immunotherapeutic targets. Wherry et al. have reported that *TOX*+ cells can express inhibitory receptors such as *CD160*, *LAG3*, *TIGIT*, and *PD-1*, and suggest that *TOX* is a major molecule which regulates the differentiation of TEX at the transcriptional and epigenetic levels (33). In our study, high level of *Nuf2* expression was significantly correlated with *TOX* and *TIM-3*, thus clarifying the potential function of *Nuf2* in the induction of TEX *via* the *TOX* and *TIM-3* pathways. This may explain the reason underlying the relationship between high *Nuf2* expression or high levels of immune cell infiltration and low survival rate in patients with HCC. This association may lead to the development of new immunotherapy for patients with HCC who do not respond to existing immunosuppressive checkpoint inhibitors. But for most immune cells and their subsets, *Nuf2* was only related to some (not all) of the biomarker genes, indicating that there is a certain specificity and selectivity in this interaction, which also provides some basis for immunotherapy in the future.

It should be emphasized that although big data analysis can comprehensively and rapidly mine potential data and functional biomolecules, various false-positive results are inevitable. Our work was mainly to provide a fast and simple method for functional genes screening, and point out a direction for future research. However, accurate conclusions need further experimental analysis and clinical verification.

## Data Availability Statement

The original contributions presented in the study are included in the article/**Supplementary Material**. Further inquiries can be directed to the corresponding author.

## Ethics Statement

The studies involving human participants were reviewed and approved by all the research data based on the bioinformatics analysis of the open resources from the TIMER, Oncomine, TCGA, Kaplan–Meier plotter, and GEPIA databases. Written informed consent for participation was not required for this study in accordance with the national legislation and the institutional requirements.

## Author Contributions

XX and XL contributed to the concept and wrote the manuscript. XX and SJ designed the experiments, performed the experiments, and analyzed the data. XX and SJ contributed equally to this study. All authors contributed to the article and approved the submitted version.

## Funding

This work was supported by the 2019 Doctor Initiation Fund of Guizhou University of Chinese Medicine (3043-043190019) and Research Initiation Foundation for Doctor of Henan Agricultural University (30602107).

## Conflict of Interest

The authors declare that the research was conducted in the absence of any commercial or financial relationships that could be construed as a potential conflict of interest.
