# Supplementary material for: Nuf2 Is a Prognostic-Related Biomarker and Correlated With Immune Infiltrates in Hepatocellular Carcinoma
Source: Front Oncol. 2021 Mar 9;11:621373. doi: 10.3389/fonc.2021.621373 (PMC7985438; doi:10.3389/fonc.2021.621373)
Supplement: Supplementary file 1 [file DataSheet_1.docx]

**Supplementary**
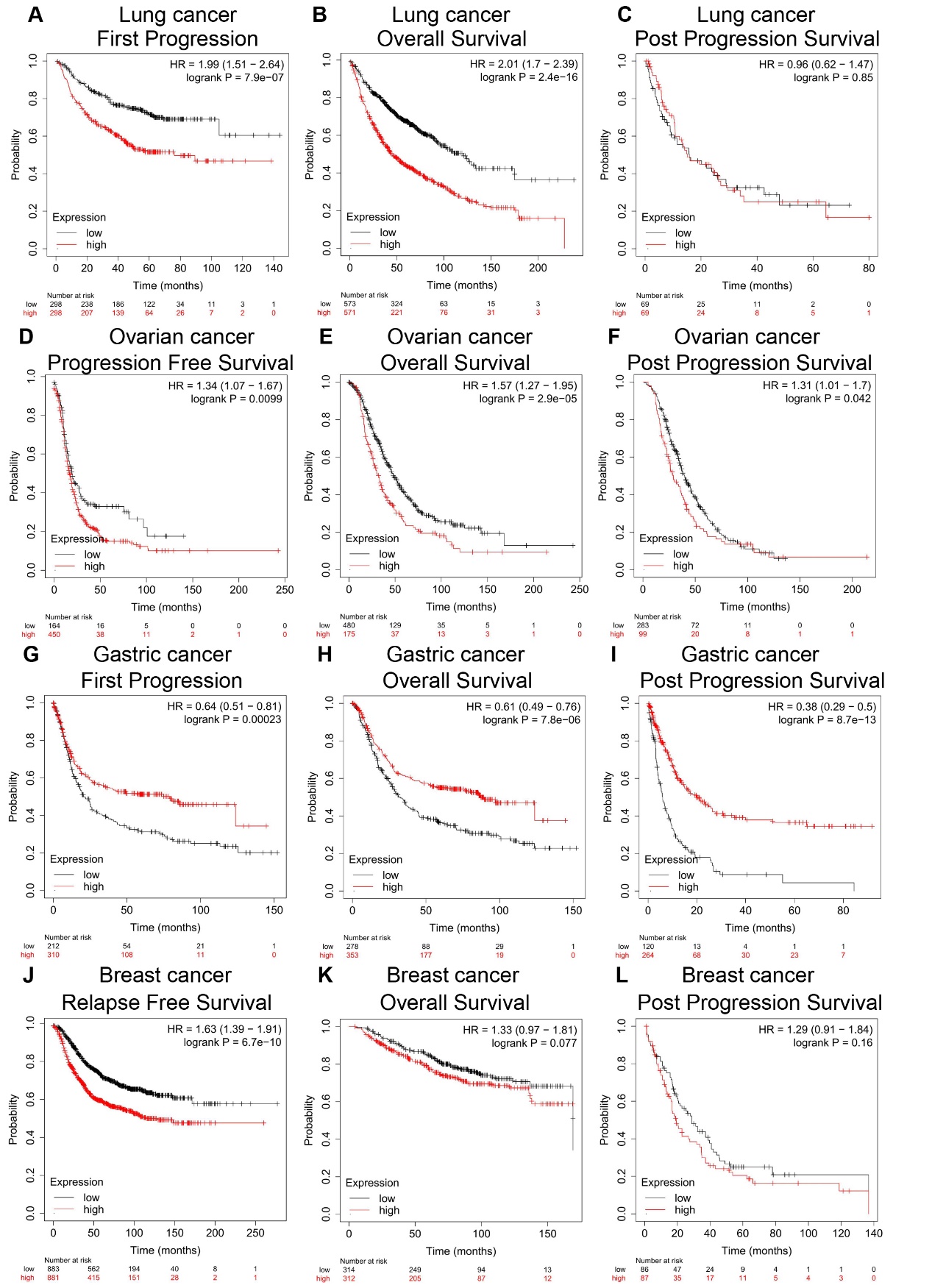


Figure S1 Correlation analysis between *Nuf2* expression and prognostic survival of cancer patients via Kaplan-Meier plotter analysis. (A-C) Lung cancer; (D-F) Gastric cancer, (G-I) Ovarian cancer; (J-L) Breast cancer.


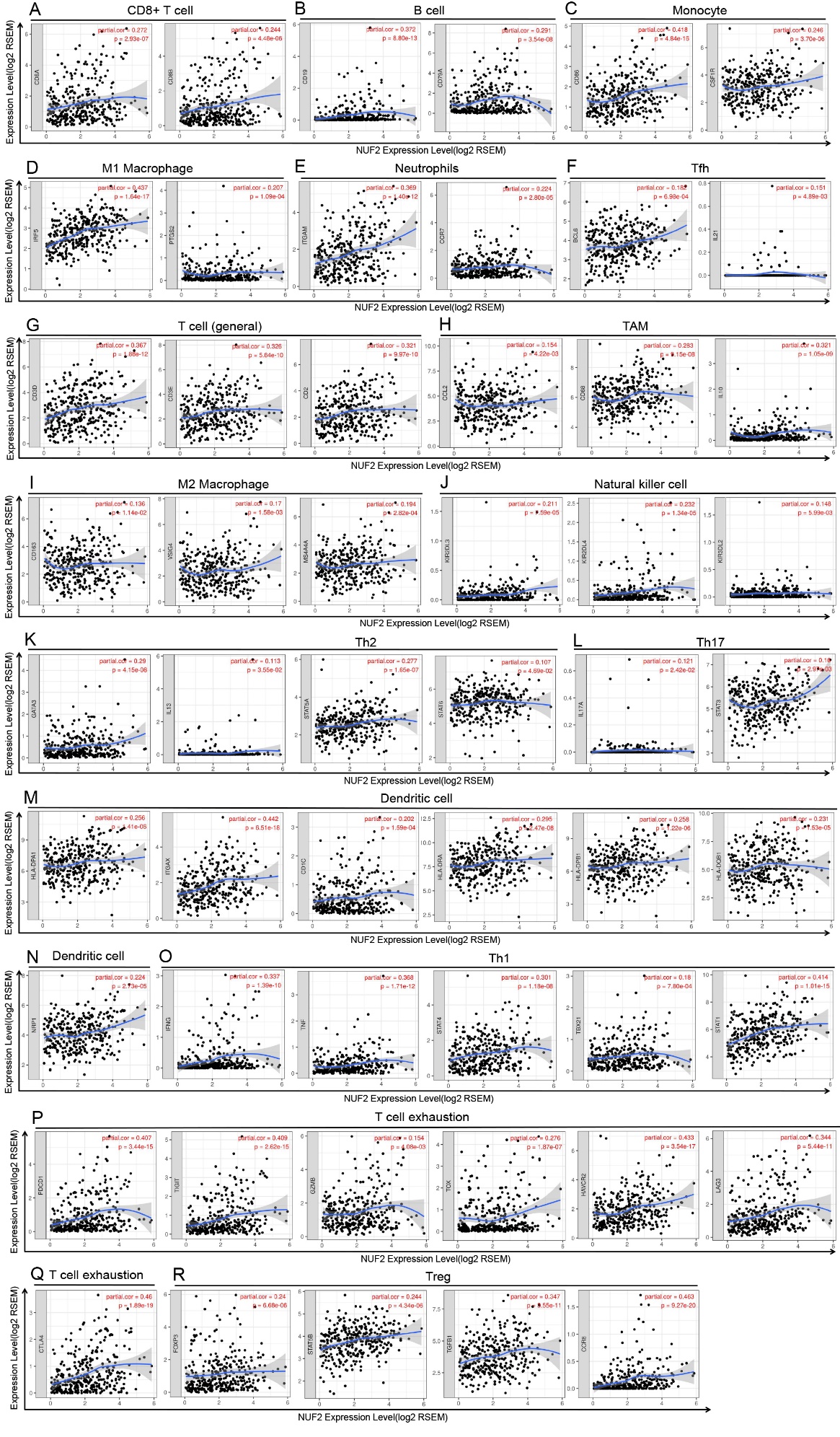


Figure S2. Correlation between *Nuf2* expression and biomarker gene expression of infiltrating immune cells in HCC analyzed via TIMER (n = 371).
